# The Lord Chief Baron and the Nottidge Case

**Published:** 1849-10-01

**Authors:** 


ART. V.—(1.) *A Remonstrance with the Lord Chief Baron, touching the case Nottidge* v. *Ridley.* By JOHN CONOLLY, M.D., Fellow of the Royal College of Physicians, Physician to the Middlesex Lunatic Asylum at Hanwell, &c. London: Churchill, 1849. 8vo. (second edition.)

(2.) *A Letter to the Lord Chancellor on the Defect of the Law regulating the Custody of Lunatics.* By CHARLES CURTON COOPER, one of Her Majesty's Counsel. London: Stevens and Morton, 1849. 8vo, pp. 15.

(3.) *Copy of a Letter to the Lord Chancellor, from the Commissioners in Lunacy, with reference to their Duties and Practice, under the Act* 8 *and* 9 *Vict., c.* 100. Ordered to be printed by the House of Commons, on the motion of Lord Ashley, M.P., and Chairman of the Commissioners in Lunacy. 8vo, pp. 12.

IN the gradual progress of knowledge and the extension of humanity, IDEAS sway to and fro with as much regularity as the movements of the pendulum. It is the great business of discussion to prevent these oscillations from becoming too violent or discursive. This observation is specially applicable to the advancement of the medical and legal management of insanity. Even at the advent of the Christian era, insane persons were held by the apostles themselves, to be "possessed of devils;" and from that time until towards the end of the last century, these unhappy creatures continued to be regarded as objects of punishment and persecution, rather than of sympathy and kindness. It would be difficult to imagine in the widest stretch of fancy, the miserable sufferings of these outcasts of society, during so many hundreds of years, whilst violence and coercion were considered to be the bounden duty of those who had the regulation of the insane. This, indeed, offers a chapter of horrors in the history of the human race, from the scrutiny of which the mind recoils with a sense of remorse and degradation. The treatment so long mistakenly pursued, developed insanity to such a fearful intensity, as we humanely hope may never in these or future times be witnessed again. The insanity of the present day, often sufficiently fearful, is mild in comparison with the malady of former days, when the chain and the gyve, the blow and the cell, were well nigh the sole medicines and ministers for the insane; when it was common enough for keepers to murder lunatics by their violence, and for madmen to turn upon their tyrants and destroy them with their own manacles. In this country, men are now living who can remember the time when, in many of our villages, the only place of restraint for the furious maniac was the stocks, or the pen for impounding stray cattle. The unfortunate lunatic was held to be something between the criminal and the beast of the field. If we look to the causes of this cruelty towards the insane, we shall find them rather in the imperfect constitution of society and in the Cimmerian darkness which so long prevailed respecting the real nature of insanity, than in any wanton cruelty on the part of the cultivators of medicine. Moreover, insanity was late in becoming strictly a medical subject. Even at the present time there exists a set of philanthropic fanatics, who claim the insane for religion, rather than for medicine, and it must be admitted that, during the worst epoch of their management, or rather mismanagement, they were the liege subjects of theology instead of the art of healing. Our own profession was not sufficiently advanced to claim its right in the teeth of ignorance and superstition, even so lately as the close of the reign of George III., of insane memory,—for it is well known that that unfortunate sovereign was more under the care of a quack divine than the regular physicians. We should wonder now to see any patient of royal blood consigned to the care of a Rev. Dr., and his cold water beds, but the parallel events actually happened only three reigns ago.

But the last decade of the eighteenth century saw the dawn of a brighter day for the insane. Even in that time of great thoughts, engines of the mind which have since shaken the world, it was a bold conception of PHILIP PINEL, who struck the fetters from some of the most violent maniacs at the Bicêtre, and proclaimed to the world that henceforth the strict enforcement of gentleness should be the great coercionary method in the management of the insane; that assuasive kindness should take the place of the bolt and the scourge—that lunatics should be received into the human family as objects of the profoundest sympathy and attention. Yes! one act of a benevolent genius established for future generations that the insane were to be treated as *patients* and not as *prisoners*. What must have been the sensations of that illustrious man after releasing some of the very strongest and fiercest maniacs from the chains which had bound them for years to the wall, men whom others were afraid to trust with liberty for a moment, lest they should rend their keepers! Pinel found that, instead of the spring of the tiger, they greeted him with tears of gratitude, and were passive and obedient as children to his commands. Pinel's was a noble experiment, performed at the risk of his own life. Humanity can never offer a sublimer spectacle than that of a good man standing between insanity and cruelty!

Since the time of Pinel, the humane idea generated in his mind has gathered strength in all civilized countries, and it has, when carried to its utmost length, produced even some excesses. Whilst we deal tenderly with these excesses, on account of their origin, it is nevertheless our duty to point them out for remedy and correction. No man really conversant with the matter, can gainsay the fact, that in this country, as in others, medical men have been the foremost amelioators of the condition of the insane. As the management and study of insanity have improved, asylums for the insane have passed from lay into medical hands, so that at the present time the treatment of insanity is almost purely in the hands of the profession. In truth, medicine receives it as an admitted truth, that PSYCHOLOGY is the very highest department of medical study, and some of the greatest minds we possess have been devoted to this most important subject. For the results, we can point triumphantly to the splendid works on insanity and other matters relating to the insane, which have been produced within the last half century—to the diminished mortality among the insane, and to the vastly-increased proportion of cures which have been effected. Yet it is a humiliating fact, that in the over-excitement of the public mind on this topic, there is evidently manifested a prevailing jealousy of the profession in all that concerns the insane. In the trials of criminals suspected of insanity, or persons undoubtedly insane, in considering the crimes committed during paroxysms of insanity; in commissions of lunacy; and in every other instance in which medicine comes before the public in connexion with insanity, this jealousy is most flagrantly apparent. The profession is treated as though it deserved punishment rather than gratitude, for its exertions in behalf of the insane. Every opportunity of blame, deserved or undeserved, is eagerly seized upon by the organs of public opinion. A society actually exists, "The Alleged Lunatic's Friend Society," holding public meetings, publishing transactions, offering premiums for anti-medical essays, the great—nay, sole object of the association being, to destroy what its members imagine to be, the existing medical despotism towards the insane. Doubtless, there are, in the profession, as in all large bodies of men, unworthy individuals, and some of these occasionally commit faults in the management of the insane, for which reprehension and punishment are due. From none do they receive them more emphatically than from their professional brethren. But such cases are too few and rare to justify the public jealousy and suspicion. No; we must attribute the major part of the public illiberality towards the profession to an excess of the benevolent sentiments for those afflicted with insanity—principally fostered and carried into execution by medical men themselves. Mixed with this, there is a natural sensitiveness on the question of the liberty of the subject, an invasion of which, by medical men, is held in great professed horror. The public mind has been so harrowed and saturated by tales of asylum atrocities, of Bedlam and St. Luke's in the olden time, that men cannot think of these places even now, save as dens of lust and barbarity. No layman who has not visited a well-conducted asylum, where kindness is the invariable rule; where restraint, or rather, *control*, is invisible; where the appearance, and not the appearance merely, of family association predominates; where an inexperienced person would scarcely distinguish the lunatics from their attendants, can fairly judge of the case as it at present stands between the medical profession and insanity. Yet every one thinks himself constituted a judge upon this matter, every one dreams of an insane person not as he is, gardening, reading the newspaper, playing at billiards or cricket, in the gymnasium, or taking unlimited exercise; but they imagine him after the pattern of Sterne's prisoner, making the dreary notch in his stick which told of a day of misery, by the one dreary ray of light allowed to penetrate his dungeon. The public mind seems drunk, if we may so express it, with humanity upon this matter. Such is the extent of this, perhaps pardonable, but certainly mischievous sensitiveness, that it cannot be tolerated even that the insane should submit to that kindly control quite compatible with non-restraint, and which is necessary to defend themselves and others from injury, and, above all, necessary to the cure of insanity. It is a disagreeable necessity that medical men should have to curb these excesses of humanity, but it is a necessity, and one to which they must submit, taking care at the same time that the real interests of the insane do not suffer. That the profession will do their duty, the public has a warranty in the past conduct of those medical men who are engaged in the treatment and elucidation of insanity. We are led to these preliminary remarks by the recent case of "Nottidge *versus* Ripley," in which the prevailing and injurious jealousy of the public towards the profession, has been rendered very prominent both by the *dicta* of the learned judge upon that occasion, and by the pretty unanimous comments of the public press. If this feeling were allowed to dominate unreproved, the humanity-mongers would soon degrade the treatment of insanity into a branch of quackery, for none would be held fit to treat the insane but those willing to bow to the prejudices of ignorance and jealousy. For ourselves, we think that our strong advocacy of the humane treatment of the insane, and our constant efforts to establish generally a safe yet gentle system of non-restraint, are too well known to render any apology from us necessary, when we undertake to remove from the public mind the antiquated and unjust notions fostered by self-elected reformers, and recently countenanced by the bench.

The strictly medical and medico-legal points of the Nottidge case may be very briefly summed up. Miss Nottidge, after exhibiting various unmistakable symptoms of unsoundness of mind, secreted herself at Charlyn, in the Agapemone of Mr. Prince. From this place she was abducted through the agency of her mother and her brother-in-law, and brought to London. She was then examined by two professional men with a view to ascertain her state of mind, and as she was found to be insane, the proper certificates were signed, and she was placed in a respectable lunatic asylum. After remaining in the asylum for a considerable time, and still preserving her delusions, she broke her word of honour and escaped from thence. In this escape, she was assisted by Mr. Prince and his agents. She was, however, re-captured, and again conducted to the asylum. After a while, her health suffering, and the state of her health being weighed against her delusions, which still existed in their full force, she was liberated most humanely, but, as it now appears, most unwisely by the Commissioners in Lunacy. Let us, for a moment, inquire into the strictly medico-legal portions of their proceedings. The medical profession certainly had nothing whatever to do with the original abduction by force from Charlyn. That was an illegal and punishable act of violence, but still an act quite distinct from her subsequent medical surveillance. But even for this illegal proceeding, the majority of sound thinking persons will find some excuse in the feelings of an aged mother, who had already lost three daughters by the same agencies, which were now entrapping the person and fortune of her eldest and weakest child. However this may be, the abduction was strictly extra-professional—the act, simply, of her own friends. The procedure of confining her to a lunatic asylum was, on the other hand, strictly formal and legal, and based upon professional proceedings. Two medical gentlemen of the highest respectability, totally unconnected with the abduction from Charlyn, certified unhesitatingly to her insanity, giving, of course, the grounds of their opinion; and she was, with perfect legality, placed in an asylum. Once formally placed there, she was under the management and jurisdiction of the Commissioners in Lunacy, who, acting under the Lord Chancellor, are the legal guardians of such persons. We have made this careful distinction between the abduction and the reception into an asylum, because the two things, have with more ingenuity than honesty, been purposely mixed up together in order to confuse the public, and excite a prejudice against the defendants in the recent action, and also against the medical profession. One point, too, has been altogether left out of sight by our diurnal and hebdomadal philanthropists—namely, the attempted abduction from the Moorcroft Asylum by the prosecutors, which was a matter quite as illegal as the original abduction from Charlyn. No unprejudiced person can say that the unfortunate lady was not more emphatically under the protection of the law at the asylum than at the Agapemone. The continuance of Miss Nottidge at Moorcroft, and also her subsequent removal was, therefore, as we have said, strictly legal, and accompanied with all the forms demanded by the law. There can be but one opinion that the only censurable point in the whole matter was her liberation and removal from all control. Far happier would it have been for all the parties concerned, excepting, of course, the financiers of the Agapemone, had she remained up to the present time either in an asylum, or in some private residence under proper control. The following observations of Dr. Conolly are most interesting and to the purpose:—

"If the judges and the members of the bar would take the trouble of visiting asylums more frequently, the medical and the legal profession would not so often be brought into collisions, in which truth is generally sacrificed to authority. If the able writers of the public press would take the same trouble, it would prevent their falling into vulgar exaggerations respecting the condition of those placed in asylums. They would all derive useful and, I think, welcome information from opportunities which would be everywhere cheerfully afforded to them. They would learn to take more comprehensive and more exact views of the nature of insanity; and would become convinced that the name of lunatic asylum ought no longer to be received as that of a place of cruelty, suffering, torture, and horror. In the last ten years, changes have taken place in them into which, if I may trust to my own observation, neither lawyers nor writers for the public have much cared to inquire. The lawyer must adhere to his definitions, and make either truth or falsehood strong as it happens; and the writer for the public must startle and amaze, and therefore he still draws on recollections of the past, or on his imagination. Thus it is seldom that either of them, when lunacy is in question, meets the medical man with candour, or on equal ground.

"All well-conducted asylums have now become places of protection, abounding in the means of diverting the thoughts, of calming morbid excitement, of soothing the depressed, of rousing the apathetic, and of restraining the lower propensities of the insane, and restoring the control of reason. The most powerful of all restraints is found to be kindness, and it would be well for mankind if, in their intercourse with one another without the walls of asylums, they imitated the forbearance exercised to those within them. The patient who was wasting his money when at large, or forming a degrading connexion, or lost in drunkenness, or wandering about, dirty and ragged, followed by the idle and mocking crowd, or vexing the quiet of many houses by night and day, and breaking the heart of sorrowing relatives, becomes, after a short residence in a good asylum, composed in manner, decent in conduct, orderly in dress; he is saved from ruin as to his property, or from impoverishing those related to him; and enjoys a degree of liberty and happiness which no other residence could afford him. To forbid the placing of such persons in asylums because they are not dangerous, can never have been your lordship's deliberate intention. It would be to forbid their being protected and cured, and to consign them to every variety of insult, and injury, and suffering, and loss."

We trust that not a doubt can remain on the mind of any person, that as far as our own profession was concerned, the most jealous stickler for the rights of the insane can have nothing whatever to complain of, as to the manner in which Miss Nottidge was originally placed in an asylum. It is the merest cant to look upon the case as one of medical persecution or despotic authority. The law was carried out fairly, and to the letter. If there was any fault therefore, it was in the constitution of the law, not in the supposed tendency of medicine to despotism. Let us, therefore, next examine into this part of the question, which removes it from the personalities of an individual case. When the Lord Chief Baron gave it as his judicial opinion, that "no person ought to be confined in a lunatic asylum unless dangerous to himself and others," he either condemned the law or its administration, or intended to do so, and he further implied a new definition of insanity, which would exclude all cases except the homicidal and the suicidal. His lordship's definition of insanity has been ably handled by Dr. Conolly in his letter, which we have no doubt the great majority of our readers have already perused with delight. This physician has shown that, in a vast number of cases of insanity, there is no tendency to homicide or suicide, but still such an absence of all moral control, on the part of the individual, as to render the assumption of some other control absolutely necessary for the peace and safety of families, and the rescue of the person affected from the commission of a variety of crimes neither homicidal nor suicidal. The forms of insanity which manifest themselves in theft without motive, in uncontrollable lust, in the assumption of every variety of titles from emperors and popes to parish paupers, and from archangels to devils, are none of them *necessarily* "dangerous," in the sense used by Sir Frederick Pollock. Almost every large asylum has its poor maniac with the delusion of boundless wealth; its rich inmate with the dread of abject poverty; persons of originally pure mind are seen indulging, as far as is allowed, in obscene and profane conversation; others, of naturally trifling habits and modes of thought, assume the style of prophets and founders of new sects in religion. Common sense, which rides even over the opinions of a judge, pronounces these persons to be mad, and science confirms the judgment, but my Lord Chief Baron's creed would not allow him to place such persons under constraint. We fear, if the large classes of insane persons, extra-homicidal and extra-suicidal, were let loose upon society, there would soon be sad work, and, in the end, the insane would be treated far more rigorously than at present.

Again, we need scarcely point out, for it has been repeated and reiterated, that a numerous class of lunatics have lucid intervals, sometimes of great length, during which their conduct and disposition are most gentle, but who on a sudden may be seized with the tendency to injure themselves or others. These sudden exacerbations of a dormant malady, cannot be foreseen, or the times of their appearance reduced to any rule, though the experienced physician, testing the delusions of the patient even in his lucidity, *can* pronounce that such and such patient, though of extremely gentle habits, is liable to this kind of paroxysm. So also, there is another class of patients who remain gentle and well behaved, while under medical control, but who on the removal of control are certainly affected with destructive tendencies. Such persons frequently beg to be defended against themselves, and implore protection of the superintendents of asylums. Critics complain of medical tyranny, but those conversant with the subject know, most painfully, that many a self-destruction, and many a murder, are committed because those unfit for liberty are released from control, and those requiring control are left to a fatal liberty too long. The Castlereaghs and the Romillys are not the only lunatics who destroy themselves from the absence of proper control. Those who, under the specious pleas of the liberty of the subject, and humanity to the insane, unduly weaken the legal constraint of the lunatic, are in reality accessories before the fact to many of the murders and suicides which, from time to time, shock society to its core.

Not only do the interests of patients, and of society, require the judicious control of all classes of the insane; but the law itself requires a further provision for it, on principles far more humane than those recently promulgated from the seat of justice. The letter signed "Ashley," which is indeed worthy the signature of that truly philanthropic nobleman, sets forth the principle of the law relating to lunacy, as it at present stands. But here we would wish to make one observation. This letter has been depreciated in certain quarters as the production of Lord Ashley alone, and therefore as carrying little weight either on a medical or on a legal topic. None could know better than such depreciators that it was the official verdict of all the commissioners, noble, legal, and medical, and as such entitled to the very highest consideration, being simply signed by Lord Ashley in his capacity of chairman of the Commissioners in Lunacy. This letter shows that the Act of Parliament under which the commissioners act as *judges* in lunacy, was intended to apply to lunatics not "dangerous," as well as those of homicidal or suicidal tendencies; and it further shows that the object and intention of the act were not merely to confine or to liberate, but to manage and to cure the insane.

"We would first refer your lordship to the Act of Parliament under which this commission is constituted, and by virtue of which persons of unsound mind are legally placed, and detained, in licensed houses and other lunatic establishments, for the treatment and cure of their disease.

"It will be observed that the object of this Act (8 and 9 Vict., c. 100) is the "Care and Treatment of Lunatics" generally, and that it is not limited to any particular class of lunatics, whether dangerous or otherwise. Indeed, the whole tenour of this and of the County Asylums Act (8 and 9 Vict., c. 126), shows that these Acts extend to lunatics of every description, and that dangerous lunatics are only occasionally noticed, where it is necessary to except and distinguish them from the rest. The Act, as set forth in its title, is "An Act for the Regulation of the Care and Treatment of Lunatics;" and the word "Lunatic" is (s. 114) defined to mean "every insane person, and every person being an idiot or lunatic, or of unsound mind;" and in the statement annexed to the order authorizing the patient's confinement, one point of inquiry is in these words: "Whether suicidal or dangerous to others;" thereby denoting that patients who are not included in that class are equally subjected to the provisions of the Act. The same observation applies to the County Asylums Act (8 and 9 Vict. c. 126), where (ss. 27 and 47, and Schedule D) dangerous lunatics are also referred to as forming part only of the body of insane persons, whose confinement and treatment in Lunatic Asylums are thereby authorized.

"The object of these Acts is not, as your lordship is aware, so much to confine lunatics, as to restore to a healthy state of mind such of them as are curable, and to afford comfort and protection to the rest. Amongst the many persons confined as being lunatics, or of unsound mind, those who are manifestly dangerous—that is to say, those who, by some overt act, have already proved themselves to be dangerous, are comparatively few in number; the far more numerous classes consisting of,—1st, Those who are sent into lunatic establishments for the purpose of treatment, with a view to the alleviation and cure of their malady; 2ndly, Those who, from disease of mind, are incapable of self-government, and who therefore require, at certain periods (or perhaps generally), the most careful supervision and control; and 3rdly, Those who are incapable of taking care of themselves or their affairs, and are likely, therefore, to sustain serious injury if left at large and unprotected."

The letter of the commissioners to the Lord Chancellor also points out those varieties of insanity indicated by Dr. Conolly, in which supervision becomes necessary, though the subject of it may not be "dangerous to themselves or others." The commissioners enumerate among them idiocy or imbecility, theomania, erotomania, nymphomania, and various other forms of moral insanity which it would be tedious and unnecessary to particularize.

Another grave question has arisen out of the Nottidge case. Granting that persons of unsound mind, not "dangerous," may properly be confined, are the defences of society against kidnapping into lunatic asylums sufficiently strong to prevent the confinement of sane persons by interested parties? We have all experienced the dread of kidnapping in early childhood. The public, made up of "children of larger growth," has taken up the notion that medical men are kidnappers, or the instruments of kidnapping, and that lunatic establishments are the receptacles for their victims. We scarcely forbore a smile when, the other day, a great organ of opinion exclaimed, "We are at the mercy of *four* signatures!" The writer was evidently trembling, or affecting to tremble, for the preservation of his own liberty in the face of doctors and commissioners. Let us see what the commissioners say about these "four signatures," and of the circumstances which should convince any reasonable person of their validity as proofs of insanity, and also the reasons why the occasions are very rare in which the immediate liberation of any person legally placed in an asylum can be ordered.

"Every person placed in confinement as a lunatic, must *primâ facie* be presumed to be insane. Before a private patient can be legally detained in any house, there must exist an order, signed by some friend or relative, two certificates from different medical practitioners, who have each separately examined the patient, and also a third certificate or statement from the medical officer of the establishment, all expressing the condition of the individual as of unsound mind, and a proper subject for confinement. It would argue great rashness and imprudence, to say the least, on our parts, to determine on the immediate, or even the very speedy liberation of a person so certified, unless we had reason to suppose that the certificates had been fraudulently obtained, or we were strengthened in our own impressions by the opinion of the medical officers having the care of the patient, that the confinement had, from the first, been improper, or that the nature of the malady was such as is usually of short duration, and that a perfect cure had been effected. Although, in a few cases of acute mania, the disorder is sometimes of short duration, yet where there exist actual delusions, the process of recovery (if ever it takes place) is slow and gradual, and the question as to the probability of cure can scarcely be determined satisfactorily until after a considerable period has elapsed."

For ourselves, we can hardly imagine such a conglomeration of villany, as that three medical men, and a "friend or relation," should be found to act together in such a hideous business as that of the premeditated confinement of a sane person, in the face of the certain and speedy exposure and punishment which must inevitably ensue, from the many means of check and detection which the law has provided. A gentleman of the long robe, Mr. C. Curton Cooper, is, however, of a different opinion; but, though desiring to examine his letter to the Lord Chancellor with all candour, we cannot say he has supported his view by any very striking or cogent arguments. Still we deem it our duty to give our readers the benefit of his opinions. We quote his opening sentences, to show how vague and shadowy his own notions are upon the matter. We italicise the words which imply so much distrust and hesitation as almost to nullify the opinions of the author :—

"A person is not merely related to or connected with an individual, but, as not unfrequently happens, he has the possession, or the control, or the management of his property, or the receipt of his income—in what character, and under what circumstances, is not material to my purpose. Now, such person being related to, or connected with the individual, has the power to have him confined as a lunatic upon his own order and statement, provided he can get the necessary medical certificate—and that, be it remembered, may be the certificate of any two apothecaries. Sometimes the certificate of one will suffice—at least, for a space. He must, of course, undertake to pay to the proprietor of the house the expenses of the individual's maintenance; but as he remains in the enjoyment (should his proceedings be successful, in the undisturbed enjoyment) of the alleged lunatic's fortune, this he most willingly does."

The strongest hypothesis put by Mr. Cooper, for he never advances beyond hypothesis or general assertions, is this:—

"There is a very *general impression* that persons are *sometimes* confined in houses licensed for the reception of lunatics, whom a jury would find to be of sound mind. My own experience *induces me to think* that such *impression* prevails not without cause. *Supposing*, however, the impression to be erroneous, it will not be denied that means should be used to remove it; and the best way of effecting this is to afford all possible facilities for ascertaining the real state of those unhappy persons. The object of these few lines is to inquire *whether*, in the law now regulating the custody of lunatics, those facilities exist. *Should* such facilities not exist—*should* that be the conclusion at which you arrive, those who have observed your lordship's public career are satisfied that no long period will elapse before the defect—*once that it is perceived by you*—is supplied."

To this passage we shall make more than one demurrer. There must be more than the certificate of two apothecaries—there must be the certificate of the responsible superintendent of the asylum, liable to the loss of reputation, the character of his establishment, and the forfeiture of his licence, in case of collusion or wrong doing. The certificates are sent to the Commissioners in Lunacy, who are bound to see and examine the patient at certain specified periods, so that in reality they must become a party to the three certificates. Independently of the commissioners, there is another inspecting power, in the districts not included under the word "metropolitan"—viz., the visiting magistrates, and it certainly requires some stretch of imagination to believe that with such checks and counter-checks false confinements are possible in the present day. We are, indeed, glad to see that Mr. Cooper himself is obliged to quote his examples from times long anterior to the present law of lunacy coming into operation. We ought to observe that the case to which he alludes, in which a maniac may be consigned to an asylum in a case of emergency upon *one* certificate, is the existence of a furious paroxysm, in which the patient is imminently dangerous if allowed to be at large.

But is it fair to consider the chances of dangerous lunatics remaining at large as well as the possibility, for we will not say probability, that the sane may be confounded with the insane. Insanity being an hereditary malady, the relations of insane persons are generally slow from motives of self-interest to admit the existence of insanity in their families. In many cases great mischief results from the pride which disguises the presence of insanity. After the admission of any person into an asylum, he may be discharged summarily by the commissioners, or by the visiting magistrates. The commissioners are lawyers and medical men of the greatest experience in insanity, and who are necessarily skilful in the highest possible degree in the diagnosis of the disease. Any attempt to hoodwink them by certificates respecting insane persons, even supposing three members of our profession could be found sufficiently villanous to act in concert in such a matter, would be hopeless and absurd. The cry about the "four signatures" is especially ridiculous. Are we not in a thousand things at the mercy of a quadruple conspiracy against property and life? were it not that there is some honesty in man, some safe-guard in society, and some dread of detection and punishment? Again, the visiting magistrates are always accompanied by a physician officially appointed to assist them in the inspection of asylums. Thus the public is not altogether defenceless against medical knaves and tyrants; on the contrary, the legislature has provided in a very remarkable manner for the prevention and detection of all misdoings in the matter of false confinement on the plea of lunacy. Not only have the commissioners and the visiting magistrates the power and the duty to restore cured and sane persons to society, but the relations of the patient may do so at any moment they may please. In the letter of the commissioners they say—

"The person signing the order for a patient's confinement (generally a relative or friend) not unfrequently, indeed, takes upon himself the responsibility of liberating a patient whilst still under a delusion, and before recovery, and the Commissioners have no right, and never attempt to interfere. The consequence of the premature discharge of a lunatic patient, however, is frequently a relapse, and should as much as possible be avoided. Even with all the caution now exercised by the medical officers of asylums, many of whom possess great experience, and have daily opportunities of watching the process of recovery, it has been found necessary to re-admit, within a short period, many patients whom they have discharged as recovered."

In legislating on the subject of lunacy, two great points are to be considered—society must be defended from the insane, and the risks incident to their improper removal from all control. On the other hand, the insane themselves are to be defended, and especial care should be taken that *sanity* should never be confounded with *insanity*. Are these matters sufficiently cared for under the present state of the law and its administration? We say boldly and truthfully, YES. If there be any leaning of the balance of justice one way or the other, we believe it to be in the opposite direction to that in which public feeling, a spurious humanity, and a jealousy of the profession, point. We believe that at the present moment, society is less guarded against the insane, than are the insane against society. In the present constitution and course of things, it is far more likely that insane persons may go free to the destruction of themselves and others, than that any person not insane should be detained, even for ever so short a space, in an asylum for lunatics. But we are not content with this expression of our opinion. We have given the data upon which we form it. Let others judge. If we are threatened with mischief, it is from LAXITY, rather than from DISCIPLINE.

